# Gramicidin A is hydrolyzed by a d‐stereospecific peptidase produced by *Bacillus anthracis*

**DOI:** 10.1111/1758-2229.13069

**Published:** 2022-04-10

**Authors:** Wendy E. Kaman, Kamran Nazmi, A. Ingrid Voskamp‐Visser, Floris J. Bikker

**Affiliations:** ^1^ Department of Oral Biochemistry, Academic Centre for Dentistry Amsterdam University of Amsterdam and VU University Amsterdam, Gustav Mahlerlaan 3004 Amsterdam 1081 LA The Netherlands; ^2^ Department of CBRN Protection Netherlands Organization for Applied Scientific Research TNO Rijswijk 2288 GJ The Netherlands

## Abstract

Previously we described the discovery of a *Bacillus* spp. specific peptidase activity related to d‐stereospecific peptidases (DSPs). The peptidase showed a strong preference for d‐leucine and d‐valine amino acids. These amino acids are present in the structure of the non‐ribosomal peptide (NRP) antibiotics gramicidin A, B and C and polymyxin E. To examine if the *Bacillus* spp. DSP‐related peptidase can hydrolyze these NRPs, the effect of gramicidin A and C and polymyxin E on peptidase activity in *Bacillus anthracis* culture supernatant was monitored. It was found that both gramicidins inhibited the DSP‐related activity in a competitive manner. MALDI‐TOF analysis revealed that upon incubation with *B*. *anthracis* culture supernatant gramicidin A hydrolyzation products appeared. This study shows that the *Bacillus* spp. specific DSP‐like peptidase was potentially produced by the bacteria to gain intrinsic resistance against NRP antibiotics. These results are of utmost importance in research towards antimicrobial resistance, whereas transfer of DSP‐related activity to other clinically relevant pathogens can be a serious threat to human health.

## Background

Soil contains a large scale of microorganisms among fungi, amoebae and bacteria, such as *Bacillus* spp. and *Actinomyces* spp. There, these microorganisms compete for limited resources such as nutrients and water. To eliminate their competitors several soil micro‐organisms produce antibiotics. Especially *Actinomycetes* spp. are well known for their intensive antibiotic production in soil. For instance, streptomycin, an aminoglycoside, was isolated from the actinomycete and soil‐inhabiting filamentous bacterium *Streptomyces griseus* (Schatz *et al*., 2005). Another source of natural occurring antibiotics comprises the class of non‐ribosomal peptides (NRPs) produced by *Bacillus* spp. (Sumi *et al*., 2015), such as polymyxin B and E (colistin), bacitracin, gramicidin A‐C and tyrocidine A‐C (Stansly and Schlosser, 1947; Toscano and Storm, 1982). These NRPs contain d‐amino acids in their structure to ensure proteolytic stability and to expand their bioactive spectrum (Nagata, 1999; Schwarzer *et al*., 2003).

Earlier we described the discovery of *Bacillus* spp. peptidase activity related to the degradation of d‐amino acids containing fluorogenic peptide substrates (Kaman *et al*., 2011). Based on the abundant presence of d‐amino acids in the peptidoglycan layer of the bacterial cell wall we were first tempted to speculate that this peptidase might play a role in bacterial cell wall synthesis and remodelling. However, recent in‐depth literature search disclosed the fact that virtually all NRP antibiotics produced by *Bacillus* spp. contain d‐amino acids (Nagata, 1999). This led to the renewed hypothesis that the previously found *Bacillus* spp. d‐stereospecific peptidase (DSP) related activity might be produced by the bacteria to hydrolyze NRP antibiotics in order to gain intrinsic resistance.

## Results and discussion

Previously, we described the discovery of DSP activity by *Bacillus* spp. (Kaman *et al*., 2011). Pinpointing substrate specificity using a fluorogenic peptide substrate library disclosed a preference for d‐leucine and d‐valine containing substrates (Kaman *et al*., 2013). Peptidase activity was detected in culture supernatants of *Bacillus anthracis*, *Bacillus megaterium*, *Bacillus cereus*, *Bacillus thuringiensis*, *Bacillus mycoides* and *Bacillus licheniformis*. In contrast, no degradation of d‐leucine/d‐valine containing substrates was found in culture supernatants of *Bacillus atrophaeus* (former *Bacillus globigii*) and *Bacillus subtilis* (Kaman *et al*., 2011).

**Fig. 1:**
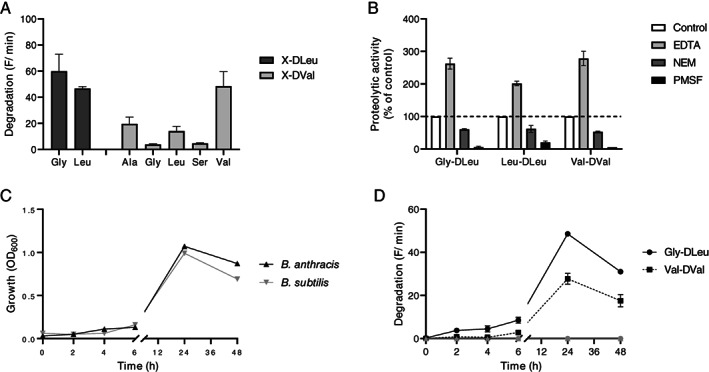
*Bacillus anthracis* produces a d‐leucine/d‐valine recognizing serine peptidase. *Bacillus anthracis* culture supernatant was incubated with all d‐leucine/d‐valine containing substrates present in the FRET‐peptide library. Proteolytic activity was defined in F min^−1^ (A). The effect of the protease inhibitors EDTA (5 mM), NEM (10 mM) and PMSF (10 mM) on the hydrolysis of the three d‐leucine/d‐valine substrates degraded with the highest efficiency. Substrate hydrolyzation without the addition of inhibitor was taken as the ‘normalized’ (100%) value (B). Proteolytic activity during growth of *B*. *anthracis* (black) and *B*. *subtilis* (grey) was monitored. Bacteria were grown for 48 h in BHI at 35°C and samples were taken to analyse growth (C) and proteolytic activity (D). Proteolytic activity was measured using the substrates Gly‐d‐Leu and Val‐d‐Val. Results are expressed as mean ± SEM (*n* = 3).

To further investigate these findings the hydrolysis of seven d‐leucine and d‐valine containing fluorogenic substrates (Kaman *et al*., 2013) was analysed using culture supernatant of *B*. *anthracis*. It was found that the two d‐leucine containing substrates, with an N‐terminal l‐glycine or l‐leucine, were degraded with comparable efficiency (Fig. 1A). Of the five d‐valine containing substrates the substrate with an N‐terminal l‐valine was degraded with the highest efficiency (Fig. 1A). The d‐valine containing substrates with an N‐terminal l‐serine or glycine were not hydrolyzed (F/min < 5) by *B*. *anthracis* culture supernatant (Fig. 1A). The culture medium itself, BHI, was found to be devoid of d‐leucine/d‐valine proteolytic activity (data not shown). These results were comparable to our earlier findings (Kaman *et al*., 2011; Kaman *et al*., 2013). Furthermore, to characterize the peptidase, we examined the effects of several (specific) protease inhibitors on the d‐leucine/d‐valine proteolytic activity. Upon addition of EDTA, a metalloprotease inhibitor, a two‐fold increase in peptidase activity was observed (Fig. 1B). This indicated that the activity of the peptidase is in a negative manner influenced by metal ions. The addition of the cysteine protease inhibitor NEM slightly affected peptidase activity. In contrast, the serine protease inhibitor phenylmethylsulfonyl fluoride (PMSF) completely inhibited d‐leucine/d‐valine proteolytic activity. These results suggest that the d‐leucine/d‐valine peptidase belongs to the family of serine proteases and that its activity is potentially regulated by metal ions. Regulation of peptidase activity by metal ions is a known mechanism. For example, the activity of the serine protease HtrA produced by the bacterium *Helicobacter pylori* is inhibited by zinc and copper ions (Bernegger *et al*., 2020). Also, Higaki *et al*. (1990) observed regulation of the activity of a serine protease by a metal switch.

To gain more insight into the functionality of the d‐leucine/d‐valine proteolytic activity, protease production was monitored during growth. *B. anthracis* and *B*. *subtilis* showed similar growth curves with a decrease in OD600 at 48 h (Fig. 1C). *B. anthracis* showed proteolytic activity which correlated with the increase in OD600. No degradation of the substrates was detected using the *B*. *subtilis* culture supernatant (Fig. 1D).

**Fig. 2:**
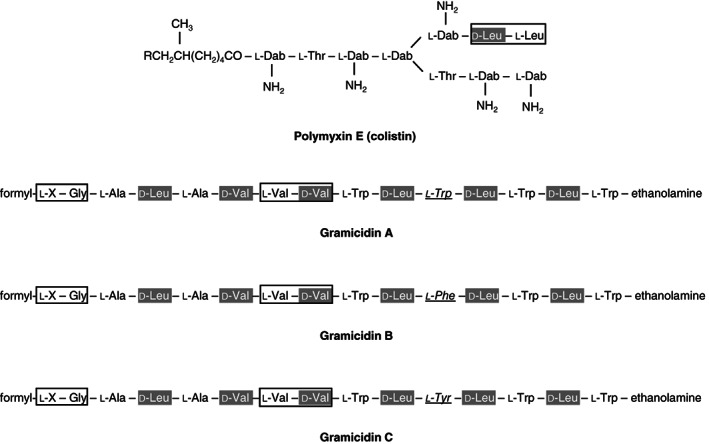
Presence of d‐leucine and d‐valine residues in the structure of NRP antibiotics. The structures of NRP antibiotics produced by *Bacillus* spp. (polymyxin E, and gramicidin A, B and C) contain the amino acids d‐leucine and d‐valine. Boxes denote the sequences corresponding to the used d‐leucine/d‐valine substrates. X is l‐valine or l‐isoleucine. Italic and underlined amino acids depict the differences between Gramicidin A, B and C.

We postulate that the d‐leucine/d‐valine proteolytic activity detected in culture supernatants of *Bacillus* spp. might lead, or contribute to, degradation and inactivation of the d‐valine/d‐leucine containing antibiotics gramicidin and polymyxin E, which contain both one or more d‐valine and/or d‐leucine residues in their structure (Fig. 2). This would be in line with our findings that *B*. *anthracis* is resistant against gramicidin A (MIC >10 μM, data not shown). In the same experiment the susceptibility of *B*. *subtilis* towards gramicidin A was determined. In contrast to the results of Wang *et al*. (2012) we found that the *B*. *subtilis* strain used in this study also had a MIC of >10 μM (data not shown). However, the secretion of a d‐leucine/d‐valine peptidase by *B*. *subtilis* to degrade d‐valine/d‐leucine containing antibiotics is unlikely, whereas this bacterium produces the d‐leucine containing antibiotic surfactin (Tojo *et al*., 2015). This suggests that *B*. *subtilis* and *B*. *anthracis* use different mechanisms to gain resistance against gramicidin A.

**Fig. 3:**
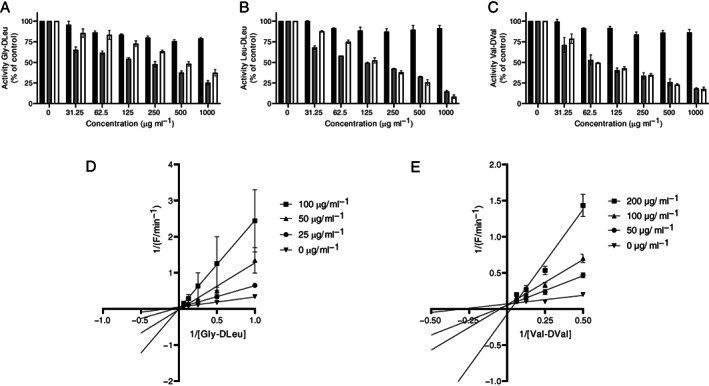
Activity of the d‐leucine/d‐valine peptidase is inhibited by gramicidin A and C in a competitive manner. The effect of varying concentrations of polymyxin E (black bars), gramicidin A (grey bars) and gramicidin C (white bars) on degradation of the FRET‐peptide substrates Gly‐d‐Leu (A), Leu‐d‐Leu (B) and Val‐d‐Val (C). Proteolytic activity without the addition of antibiotics was used as reference (100%) value. Lineweaver–Burk plot for the inhibition of Gly‐d‐Leu (D) and Val‐d‐Val (E) degradation by gramicidin A. Results are expressed as mean ± SEM (*n* = 3).

**Fig. 4:**
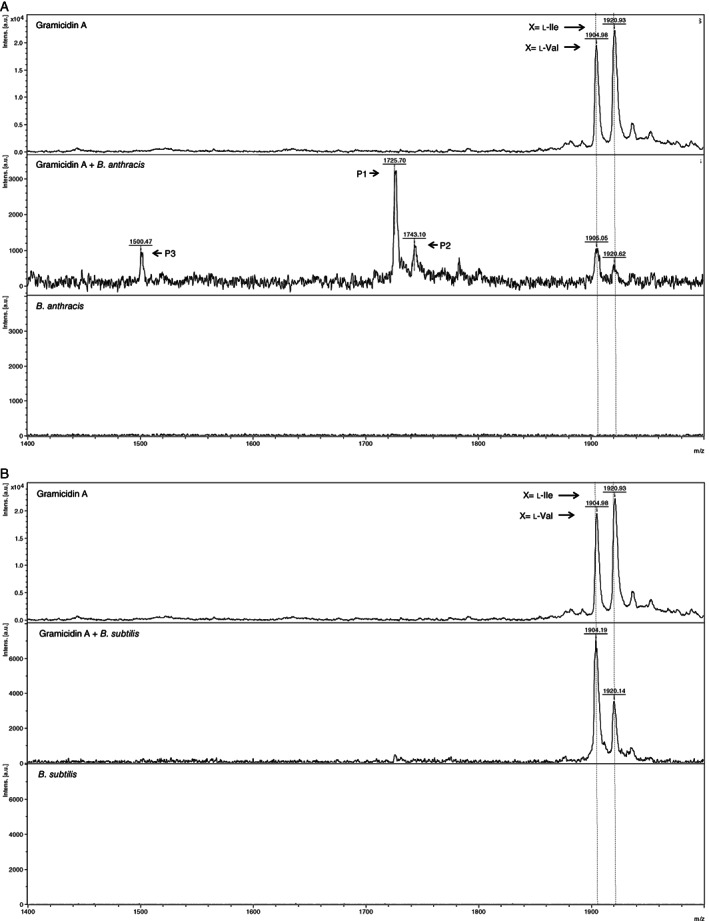
Hydrolyzation of gramicidin A is degraded by peptidase activity present in *B*. *anthracis* culture supernatant. MALDI‐TOF analysis of gramicidin A incubated with *B*. *anthracis* (A) or *B*. *subtilis* (B) culture supernatant. Culture supernatants and gramicidin A alone were used as negative controls. Gramicidin A degradation products are depicted in P1‐3.

To check the aforementioned hypothesis the effect of gramicidin A, C and polymyxin E on the activity of the d‐leucine/d‐valine peptidase was examined. Gramicidin B, however, was not included, whereas this antibiotic was not commercially available. It was found that both gramicidin A and C inhibited the d‐leucine/d‐valine proteolytic activity on all tested substrates (Fig. 3A–C). In contrast, no inhibition of proteolytic activity was observed when polymyxin E was added to the peptidase assay. This might be related to the fact that in the structure of polymyxin E N‐terminal from d‐leucine the unnatural amino acid l‐α,γ‐diaminobutyric acid (l‐Dab) is present (Fig. 2). l‐Dab is not commonly recognized by peptidases and thereby potentially protects polymyxin from hydrolyzation by the d‐leucine/d‐valine peptidase (Lu *et al*., 2020). The type of inhibition by gramicidin A was studied using two fluorogenic peptide substrates in which a comparable amino acid motif was present as in gramicidin A; valine‐d‐valine and glycine‐d‐leucine (i.e. glycine‐isoleucine in gramicidin A) (Fig. 2). Analysing the enzyme kinetics the Lineweaver–Burk plot showed that gramicidin A inhibits d‐leucine/d‐valine proteolytic activity in a competitive manner (Fig. 3D and E). This indicates that gramicidin A competes with the d‐leucine/d‐valine substrates for binding to the *Bacillus* spp. peptidase and suggests that gramicidin A might be the natural substrate for the d‐leucine/d‐valine peptidase produced by *B*. *anthracis*. To further investigate this hypothesis gramicidin A was incubated with culture supernatant of *B*. *anthracis*. As negative control culture supernatant from *B*. *subtilis*, a *Bacillus* spp. which lacks d‐leucine/d‐valine peptidase production, was used (Kaman *et al*., 2011). MALDI‐TOF analysis revealed that gramicidin A (*m/z* 1905 *X* = l‐Val/*m/z* 1921 *X* = l‐Ile) was indeed degraded by *B*. *anthracis* culture supernatant (Fig. 4A). No degradation products were observed when gramicidin A was incubated with culture supernatant of *B*. *subtilis* (Fig. 4B). The sizes of the degradation products in the gramicidin A sample incubated with *B*. *anthracis* supernatant were *m/z* 1726 (P1/*X* = l‐Val), *m/z* 1743 (P2/*X* = l‐Ile) and *m/z* 1501 (P3/*X* = unknown). The peak sizes of P1/P2 correspond with C‐terminal hydrolyzation of gramicidin A between d‐leucine (L14) and l‐tryptophan (W15). To confirm this cleavage position and to unravel the identity of peak P3 additional experiments are needed. Whereas no peaks were observed in the *Bacillus* supernatant alone samples, we can conclude that the peaks present in the gramicidin A/*B*. *anthracis* sample originate from gramicidin A (Fig. 4A and B).

That *Bacillus* spp. are able to protect themselves against the antimicrobial effects of NRP antibiotics produced by hydrolyzation has already been described by others (Li *et al*., 2018; Yin *et al*., 2019). The hydrolyzation mechanism discovered in the study of Li *et al*. is in line with the results of our study; using a networking‐associated genome‐mining platform Li *et al*. found two d‐stereoselective peptidases produced by bacteria of the order of Bacillales; TriF (*Paenibacillus polymyxa*) and BogQ (*Brevibacillus laterosporus*). Additionally, like BogQ, the d‐leucine/d‐valine peptidase described in this study belongs to the family of serine peptidases. According to the MEROPS database, the genome of *B*. *anthracis* contains 15 known or putative serine peptidases which are absent in the genome of *B*. *subtilis* (Table S1). Six of these *B*. *anthracis*‐specific serine peptidases belong to the subfamily S12, of which BogQ is a member. Potentially the degradation of the d‐leucine/d‐valine substrates is related to the activity of one of these six S12 peptidases but additional research is needed to unravel the identity of the DSP peptidase described in this study.

Our results show that a range of *Bacillus* spp., among which the biowarfare associated pathogen *B*. *anthracis*, produce a peptidase with DSP related activity. That also other bacteria in the order of Bacillales can produce NRP hydrolyzing DSPs was to be expected; 403 putative peptidases with similar domain compositions to BogQ are present in the order of Bacillales (Li *et al*., 2018). The DSP‐related activity we discovered in 2011 differs from BogQ and TriF in d‐amino acid preference. Where TriF hydrolyzes the C‐terminal side of aromatic d‐amino acids and BogQ cleaves peptides at the C‐terminal side of cationic d‐amino acids the DSP from *B*. *anthracis* shows a preference for d‐leucine and d‐valine (both uncharged and hydrophobic) residues. However, the exact cleavage preferences of the *B*. *anthracis* DSP, its identity and if the observed hydrolyzation of gramicidin A leads to inactivation of the antibiotic remain to be elucidated.

The observation that different types of DSPs, with a variety in recognition sequences, are produced by different bacteria of the Bacillales order is of utmost importance in research towards antimicrobial resistance. DSPs can potentially be transferred to other, clinically relevant, pathogens and thereby be a serious threat to human health. Additionally, the existence of these dsps should be taken into account when new antibiotics are designed and for example d‐amino acids are added to their structure to improve stability (Molhoek *et al*., 2011; Bann *et al*., 2019).

## Methods

### Bacteria

*Bacillus anthracis* Vollum (NCTC 10340) and *Bacillus subtilis* (ATCC 6051) were grown overnight in 15 ml Brain Heart Infusion (BHI) medium (BioTrading, Mijdrecht, The Netherlands) at 35°C (200 rpm). For the growth curve experiment bacteria were grown in 10 ml BHI for 48 h at 35°C (200 rpm). Samples were taken at 0, 2, 4, 6, 24 and 48 h and absorbance was measured at 600 nm using a spectrophotometer (Ultrospec10, Amersham Biosciences, UK). Next, the bacteria were pelleted by centrifugation for 20 min at 3250*g*. Supernatant was filtered through a 0.22 μM filter (Millipore, Amsterdam, The Netherlands) and sterility was verified by plating 1/10 of the stock on TSA plates and subsequently incubation for 7 days at 35°C. Culture supernatant was stored at −20°C until sterility was confirmed.

### Peptidase assay

Proteolytic activity was determined, as described earlier (Kaman *et al*., 2011). Fluorescence was read for 60 min at 2 min intervals using a fluorescence microplate reader (FLUOstar Galaxy, BMG Laboratories, Offenburg, Germany) with an excitation wavelength of 485 nm and an emission wavelength of 530 nm. Peptidase activity was defined in fluorescence per minute (F min^−1^). BHI medium was used as negative control. Characterization of the peptidase was performed by the addition of specific protease inhibitors to the assay; 5 mM EDTA, 10 mM N‐ethylmaleimide (NEM) or 10 mM PMSF. None of the protease inhibitors influenced the pH of the reaction mixture. To determine the effect of d‐leucine/d‐valine containing antibiotics on proteolytic activity, *B*. *anthracis* culture supernatant was incubated with 16 μM FRET‐peptide substrate in the presence of varying concentrations of polymyxin E, gramicidin A and gramicidin C from *Brevibacillus brevis* (all purchased from Merck Life Science, Amsterdam, The Netherlands). Peptidase activity was defined as a percentage of the control (proteolytic activity without antibiotics). To determine the type of inhibition (competitive or non‐competitive) 0–200 μg ml^−1^ gramicidin A was incubated with 1–16 μM FRET‐peptide substrate. The initial velocity was plotted against the substrate concentration using the Michealis–Menten equation and GraphPad Prism8 software. All measurements were performed in triplicate, and data are presented as mean ± SEM.

### MALDI‐TOF analysis

Culture supernatants of *B*. *anthracis* and *B*. *subtilis* were incubated with 1 mg ml^−1^ gramicidin A for 72 h at 37°C. Culture supernatant without gramicidin A and 50 mg ml^−1^ gramicidin A in water were used as controls. Following incubation the samples were purified using Pierce C18 ZipTips (Thermo Fisher Scientific, Breda, The Netherlands) according to the manufacturer's instructions. Briefly, ZipTips were preconditioned with 10 μl 100% acetonitrile and subsequently washed three times with 10 μl 10% (vol./vol.) acetonitrile in water. After the application of 10 μl sample the ZipTips were again washed three times with 10 μl 10% (vol./vol.) acetonitrile in water followed by elution with 10 μl 80% (vol./vol.) acetonitrile in water. The purified samples were analysed using MALDI‐TOF (Bruker Daltonik GmbH, Bremen, Germany).

## Supporting information


**Table S1.** List of serine peptidases present in the genomes of *B*. *anthracis* and *B*. *subtilis* (Rawlings, N.D., Barrett, A.J., Thomas, P.D., Huang, X., Bateman, A. & Finn, R.D. (2018) The MEROPS database of proteolytic enzymes, their substrates and inhibitors in 2017 and a comparison with peptidases in the PANTHER database. Nucleic Acids Res 46, D624‐D632).
